# Copper Chelator Induced Efficient Episodic Memory Recovery in a Non-Transgenic Alzheimer’s Mouse Model

**DOI:** 10.1371/journal.pone.0043105

**Published:** 2012-08-21

**Authors:** Johnatan Ceccom, Frédéric Coslédan, Hélène Halley, Bernard Francès, Jean Michel Lassalle, Bernard Meunier

**Affiliations:** 1 Université de Toulouse, UPS, Centre de Recherches sur la Cognition Animale, Toulouse, France; 2 CNRS, Centre de Recherches sur la Cognition Animale, Toulouse, France; 3 Palumed, Castanet-Tolosan, France; 4 Laboratoire de Chimie de Coordination du CNRS, Toulouse, France; The Mental Health Research Institute, University of Melbourne, Australia

## Abstract

Alzheimer’s disease (AD) is a neurodegenerative syndrom involving many different biological parameters, including the accumulation of copper metal ions in Aβ amyloid peptides due to a perturbation of copper circulation and homeostasis within the brain. Copper-containing amyloids activated by endogenous reductants are able to generate an oxidative stress that is involved in the toxicity of abnormal amyloids and contribute to the progressive loss of neurons in AD. Since only few drugs are currently available for the treatment of AD, we decided to design small molecules able to interact with copper and we evaluated these drug-candidates with non-transgenic mice, since AD is mainly an aging disease, not related to genetic disorders. We created a memory deficit mouse model by a single icv injection of Aβ_1–42_ peptide, in order to mimic the early stage of the disease and the key role of amyloid oligomers in AD. No memory deficit was observed in the control mice with the antisense Aβ_42-1_ peptide. Here we report the capacity of a new copper-specific chelating agent, a bis-8-aminoquinoline PA1637, to fully reverse the deficit of episodic memory after three weeks of treatment by oral route on non-transgenic amyloid-impaired mice. Clioquinol and memantine have been used as comparators to validate this fast and efficient mouse model.

## Introduction

Alzheimer’s disease (AD) is a devastating neurodegenerative disease affecting almost 50% of people older than 85 years [1]. Less than 5% of the cases are due to a genetic disorder and more than 95% are sporadic cases. The disease is mainly related to the progressive loss of specific neurons in brain due to an oxidative stress [2]. Redox active metal ions, in particular copper, are mediating the oxidative stress and the toxicity of Aβ amyloids, since the observed concentrations of copper ions in amyloid plaques in AD-brains can be as high as 400 mM, i.e. four times higher than in normal brains [3]. The activation by endogenous reductants of the excess of copper ions in amyloid plaques induces the catalytic generation of reactive oxygen species (ROS) [4]–[7]. The tau pathology is also related with a chronic exposure of an excess of copper ions that selectively dysregulates cdk5, one of the two major kinases associated with abnormal tau phosphorylation in the brain [8].

Considering the negative effects of the trapping of copper ions by amyloids, Bush and coworkers developed clioquinol (CQ), a copper chelating agent previously used as anti-diarrhea drug, up to a phase-II clinical trial for the treatment of AD patients [9]. Clioquinol is also able to transport copper within metal-deficient neurons and has been considered as a ionophore entity with chaperone-like properties [10]. The same group promoted the development of a new hydroxyquinoline derivative (PBT2). This metal chelating agent is able to restore cognition in transgenic mice [11] and the first report on a phase-II indicated that this drug-candidate has a noticeable efficacy at 250 mg daily doses [12].

**Figure 1:**
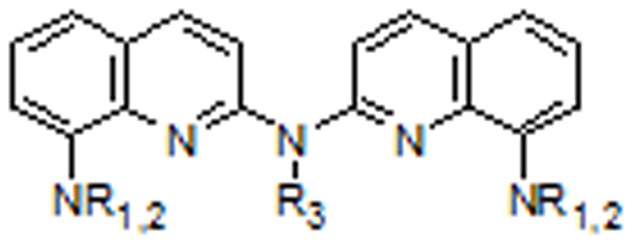
General structure of bis-8-aminoquinolines.

We decided to tackle the modulation of copper trafficking and homeostasis within aging brain with bis-chelating agents able to generate tetradentate copper complexes with the four Cu-binding sites within the same ligand (two clioquinol entities are necessary for a full complexation of copper). The low yield of the chemical synthesis of a first series of cyclic bis-phenanthroline ligands [13] prompted us to design new bis-chelating ligands, based on 8-aminoquinoline motifs, easy to prepare and selective for copper chelation (Figure 1) [14]–[17].

These bis-chelating ligands have an affinity for copper(II) that is 3 to 4 order of magnitude higher than that of 8-hydroxyquinoline monomers like clioquinol and they are also more efficient for solubilizing Aβ-peptides and as inhibitors of the H_2_O_2_ produced by Cu-amyloids activated by ascorbic acid.

One of these 8-aminoquinolines, PA1637, has been selected among this series of bis-quinolines due to its high selectivity for copper(II) chelation with a log*K*_aff_ value = 17.9 and its incapacity to complex zinc ions. This affinity constant is above the dissociation constant established for the copper-binding domain of amyloid peptides for Cu(II) and Cu(I) which are in the pico and femto molar range, respectively [18], [19]. The absence of chelation for zinc by PA1637 is a positive factor since a subacute myelo-optic neuropathy of the clioquinol-zinc chelate has been evidenced by Arbiser et al. [20].

The pre-clinical evaluation of AD drug-candidates is highly challenging since there are no evident animal models in terms of efficiency and validity. Transgenic models have been used to evidence the positive effects of the copper-complexing agent tetrathiomolybdate to attenuate amyloid pathology in Tg2576 mice (*21*) and to improve learning and memory deficits by PBT2 [12]. Adlard et al. also showed that PBT2 enhanced dendritic spine density and synaptic protein levels in hippocampus of Tg2576 mice [22]. Nevertheless, transgenic mice are a slow and expensive model for the screening and the evaluation of new molecules (experiments require 6 to 16 months old mice). Moreover, they are not highly predictive because AD is mainly an aging disease, not related to genetic disorders and also because none of these transgenic mice fully recapitulates the AD pathology [23]. Due to the lack of a fast and predictive animal model, many drug-candidates failed in phases II or III of human clinical trials. A survey of the literature suggested that in rodents, the amyloid (Aβ)-related neuropathological features of AD might be mimicked by intra-cerebroventricular (icv) or intra-cerebral injections of Aβ peptides of different lengths (reviewed in ref [24]), whereas systemic inoculation yielded no detectable induction of cerebral amyloidosis in APP23 transgenic mice [25]. Although various attempts were realized with peptides of different length, in the mouse, most of the studies using the Aβ_1–42_ human peptide showed impaired cognitive functions early (2 to 10 days) after icv injection [26], [27]. These studies suggested also that the effects the Aβ_1–42_ peptide could be induced after a single icv injection. For instance, it has been shown that between 1 and 29 days after a single icv Aβ_1–42_ injection, mice displayed impaired performances in passive avoidance, alternation behavior and spatial learning along with a decrease in acetylcholine levels in the cortex and an increase of immunoreactivities of the astrocyte marker GFAP and of interleukine-1β in the hippocampus [26]. The proposal that small soluble oligomers in the aggregation process confer synaptic dysfunction whereas large, insoluble deposits might function as reservoirs of the bioactive oligomers [28] could account for this fast loss of episodic memory after a single icv injection of small amyloids. Recent studies confirmed that Aβ_1–42_ are prone to highly aggregate as plaques and to penetrate neurons as oligomers and activate p53, enhancing neuronal apoptosis [29].

Therefore we decided to develop a « fast murine screening model » by using of non-transgenic mice suffering from cognitive deficits induced by the intracerebroventricular (icv) injection of Aβ_1–42_ oligomers that are considered as a causative agent in AD associated with oxidative stress and aging [30], [31].

Episodic memory deficits are a prominent feature of AD. In the present study, these deficits were assessed in control and treated mice, using a well-validated and automated learning procedure, contextual fear conditioning (CFC), that requires intact declarative memory that is affected in early-stage AD patients, and presents valuable methodological advantages including intrinsic validity and reliability [32]–[34].

Here we report the capacity of PA1637, a tetradentate copper-chelator, to fully reverse the cognitive deficits of Aβ_1–42_ injected mice after a three-week treatment by oral administration or intraperitoneal (i.p.) injection. Clioquinol was used as reference compound.

## Results

### Generation of a Memory Deficit in Mice by a Single icv Injection of Aβ_1–42_ Peptide

**Figure 2:**
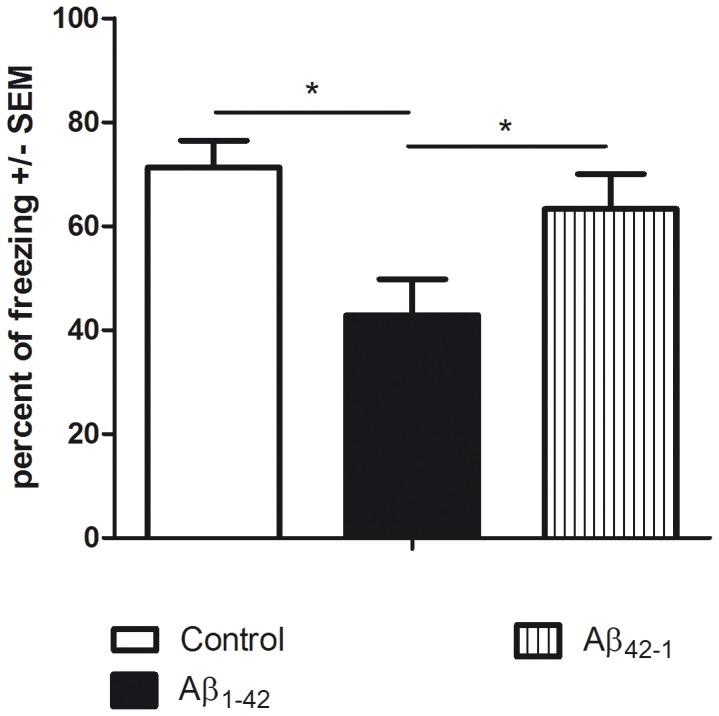
The Aβ_1–42_ injection model. Memory performances during the contextual fear memory assay show a significant variation [F(2,11) = 5.044; p = 0.027] due to the treatment factor (R^2^ = 0.478). Control mice receiving DMSO (icv) exhibited standard levels of freezing scores. The icv injection of Aβ_1–42_ resulted in a significant decrease of freezing responses compared to controls (p = 0.011) whereas the antisense Aβ_42−1_ group (p = 0.042) did not differ from controls (p = 0.409).

To fulfill the requirements (“fast” and “predictive”) of a mouse model devoted to the screening of new molecules in the field of Alzheimer’s disease, we compared the effects of Aβ_1–42_ peptide icv injections made in the lateral ventricles, with those of Aβ_42−1_ antisense peptide used as a control (Figure 2).

Mice receiving a single icv injection of the Aβ_1–42_ peptide (prepared as short oligomer, see the Material and Methods Section) showed a significant freezing impairment corresponding to a meaningful episodic memory deficit after a 21-day period (Figure 2: percentage of freezing score = 39.5%, filled black bar). On the other hand, as expected, mice receiving by icv the antisense peptide Aβ_42−1_ presented no significant freezing impairment compared to controls. Histological controls performed 50 days and 3, 6 or 9 months after the initial icv injection of the Aβ_1–42_ peptide indicated the absence of plaques that can be considered as the characteristic of the late stage of the disease. Consequently, this model based on a single injection of amyloid oligomer can be regarded as a model of the early stage of the disease.

### Oral Administration of PA1637 Restores Cognitive Deficits Induced by Aβ_1–42_ icv Injection

Having in hands this mice model, we decided to investigate the activity of PA1637, a specific copper chelating agent that might be able to restore the regulation of copper circulation since the injection of Aβ_1–42_ peptide within the brain of mice is creating a memory deficit (Aβ_1–42_ peptide can be consider as a trap for copper ions, creating consequently a depletion of copper ions in neurons).

**Figure 3:**
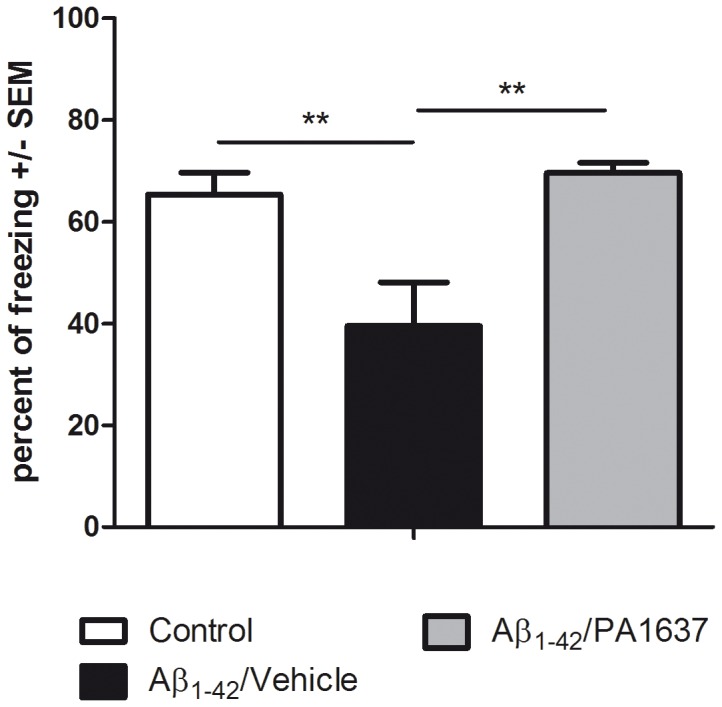
Effect of PA1632 oral administration on episodic memory deficit. Memory performances during the contextual fear memory assay show a significant variation [F(2,12) = 8.114; p = 0.006] due to the treatment factor (R^2^ = 0.575). Control mice receiving a mixture of DMSO (icv) and vehicle (oral) exhibited standard levels of freezing scores. The icv injection of Aβ_1–42_ (Aβ_1–42_/vehicle group) resulted in a highly significant decrease of freezing responses compared to controls (p = 0.008). PA1637 (Aβ_1–42_/PA1637) improved significantly the freezing scores of mice having received Aβ_1–42_ (p = 0.003).

When mice were orally treated with PA1637 three times per week at 25 mg/kg (8 doses in total), we observed no loss of cognitive deficit as depicted in Figure 3. After treatment, it should be noted that the freezing score is highly comparable with that of the control experiments (69.5% of freezing score, grey bar, compared to controls 65.3% of freezing score, open bar).

In order to validate this non-transgenic mouse model, we decided to compare the chelator PA1637 with clioquinol and memantine (MEM), a drug currently used for the treatment of severe Alzheimer’s disease [35], [36]. Comparisons were performed by injecting drugs by i.p. route to bypass the different oral bioavailabilities between these three molecular entities.

### Comparative Effects of PA1637 and Clioquinol (with i.p. Injections)

**Figure 4:**
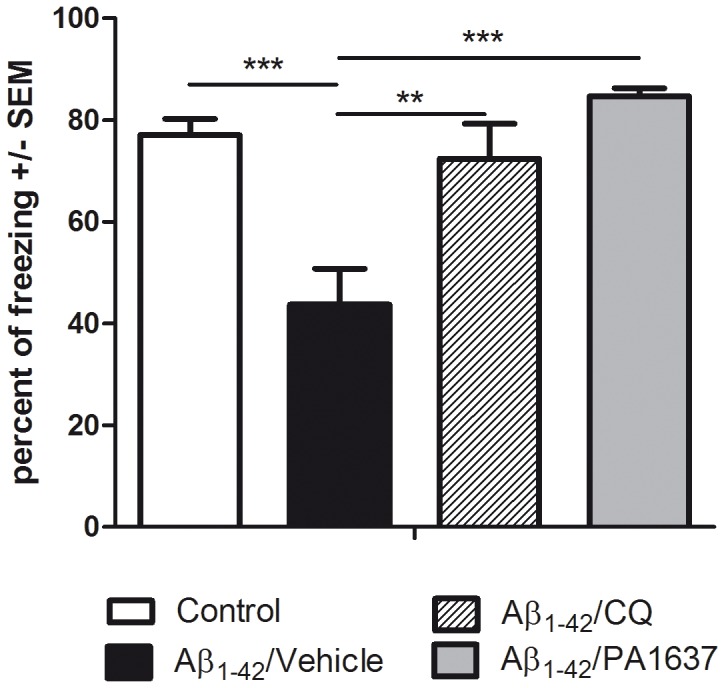
Comparative effects of PA1637 and clioquinol on episodic memory deficit (i.p. administration). Memory performances during the contextual fear memory assay show a significant variation [F(3,16) = 13.846; p<0.001] due to the treatment factor (R^2^ = 0.722). Control mice receiving a mixture of DMSO (icv) and vehicle (i.p.) exhibited standard levels of freezing scores. The icv injection of Aβ_1–42_ (Aβ_1–42_/vehicle group) resulted in a highly significant decrease of freezing responses compared to controls (p = 0.001). Clioquinol (Aβ_1–42_/CQ) improved significantly the freezing scores of mice having received Aβ_1–42_ (p = 0.002). The PA1637 molecule (Aβ_1–42_/PA1637 group) proved even more efficient (p<0.001) although the difference with the clioquinol group was only marginally significant (p = 0.08).

Again, the non-treated mice that received a single icv injection of Aβ_1–42_ showed an episodic memory deficit after a 21-day period (Figure 4: percentage of freezing = 43.7%, filled black bar) compared with 77% for controls (open bar). This result is comparable to those observed in the previous experiment (Figure 3), indicating the robustness of the assay. Both drugs were injected by i.p. route at a dose of 25 mg/kg (8 doses in total). Clioquinol with a percentage of freezing equal to 72.4% (diagonally striped bar) was slightly less efficient than PA1637 (84.7%, grey bar).

### Data with Memantine Support the Robustness of the Model

**Figure 5:**
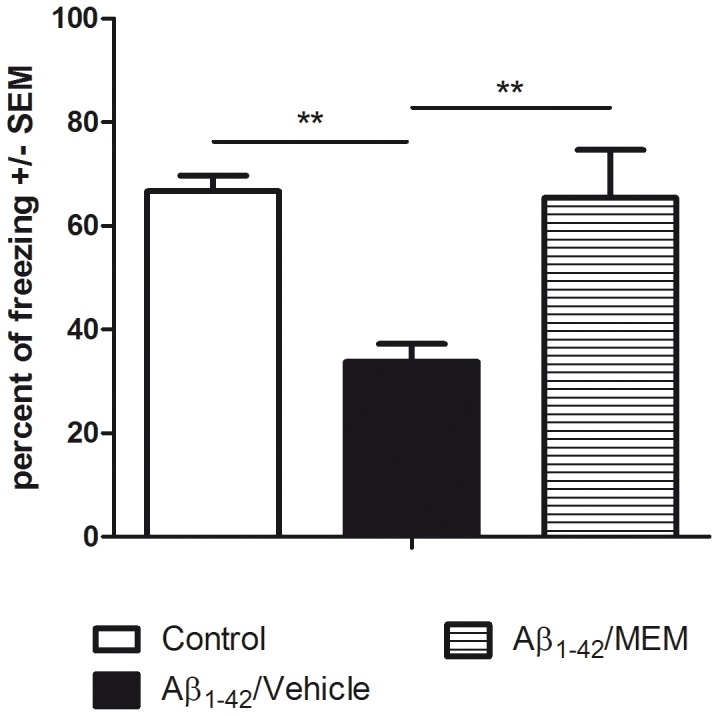
Validation of the Aβ_1–42_ injection model with memantine. Memory performances during the contextual fear memory assay show a significant variation [F(2,11) = 8.582; p = 0.006] due to the treatment factor (R^2^ = 0.609). Control mice receiving a mixture of DMSO (icv) and vehicle (i.p.) exhibited standard levels of freezing scores. The icv injection of Aβ_1–42_ (Aβ_1–42_/Vehicle group) resulted in a highly significant decrease of freezing responses compared to controls (p = 0.005). Memantine (Aβ_1–42_/MEM group) improved significantly the freezing scores of mice having received Aβ_1–42_ (p = 0.004).

When “Aβ_1–42_ mice” were treated with memantine, an uncompetitive NMDA (*N*-methyl-*D*-aspartate) receptor antagonist active in β-amyloid-enhanced excitotoxicity [35], [36], we also observed a marked impairment of freezing scores in Aβ_1–42_/vehicle mice (Figure 5: freezing score = 33.7%, filled black bar) compared to controls (66.6%, open bar). Memantine counterbalanced efficiently the memory impairment observed in Aβ_1–42_ mice (65.4%, horizontally striped bar). The fact that memantine had a positive effect as memory protector in this non-transgenic mouse model is highly supportive of the robustness of this animal model.

### Comparative Toxicity of PA1637 and Clioquinol

We determined the lethal dose of PA1637 on the same C7BL/6J mice in comparison with clioquinol (LD_50_ value: dose killing 50% of mice). The LD_50_ values by oral route were above 450 and equal to 100 mg/kg for PA1637 and clioquinol, respectively, and by i.p. injection these values were equal to 400 and below 50 mg/kg for PA1637 and clioquinol, respectively. The safety window is highly in favor of PA1637 in both cases. In addition, no ocular toxicity was observed with PA1637, which was not the case with mice treated with clioquinol. Such ocular toxicity was documented in the literature as a subacute myelo-optico-neuropathy (SMON) (see ref [37] for neurologic disorders generated by clioquinol in humans and animals).

## Discussion

The results of the present study clearly show that a single icv injection of Aβ_1–42_ oligomers generates episodic-like memory deficits in mice after a 21-day delay. The presence of small amounts of that amyloid peptide under its pathogenic form in the brain of adult mice induced memory impairments ascribable to defective consolidation or defective recall of an episodic representation. Therefore, our results suggest that deleterious effects of the Aβ_1–42_ peptide could impair the functioning of the hippocampal structure, reflecting typical features of human memory deficits observed in AD, and thus establishing the validity of this murine model of amyloid pathology. As working hypothesis, one can consider that the injection of the pathogenic Aβ_1–42_ peptide is disturbing the copper circulation by trapping a large amount of copper ions, and, as a consequence, is creating probably a deficit of copper for metalloenzymes that need this cation as essential element of their active sites. Because of its high affinity for copper ions, PA1637 is probably able to extract these ions from amyloid peptides and will facilitate the normal circulation of copper ions [38], [22]. In addition, PA1637 as well as other bis-quinoline derivatives is able to inhibit the aggregation of amyloid peptides [10], [14], [39].

Moreover, this non-transgenic mouse model presents the additional advantages of being fast, easy to implement, reliable and reproducible in independent experiments as indicated by low standard errors. During the conditioning session, irrespective to the applied treatment, mice showed similar initial levels of freezing and behaved similarly in response to the unconditional stimulus, displaying a large increase of freezing in response to the electric shock (see the absence of confusing effects of treatments during learning experiments in the Materials and Methods Section). Consequently, the different treatments did not induce variations of stress or activity during the training session. Furthermore, reactivity and short term memory of mice conditioned using the single trial training procedure were not impaired by the pharmacological treatments since mice of the different groups exhibited similar levels of freezing during the 30 sec period following the administration of the electric shock. On the other hand, during the contextual-fear memory assay that occurred 24 h after conditioning, Aβ_1–42_ mice showed impaired freezing, revealing a meaningful episodic memory deficit. The validity of the presently described Aβ_1–42_ icv model is also established by the positive effect of three different molecules, two chelators, PA1637 and clioquinol, and memantine, a NMDA antagonist to inhibit the loss of episodic memory. Such non-transgenic mice model should not be considered as a complete AD animal model, but as a useful tool for rapid screening of drug-candidates. Additional studies will be performed in the future to evaluate the limits of this amyloid model.

In conclusion, the present results establish the validity of this murine model of amyloid pathology as a “fast and predictive model”. We established that a new specific copper-modulating agent PA1637 is able to fully reverse the deficit of episodic memory after only three weeks of treatment by oral route using this non-transgenic mouse model. PA1637 can be considered as a drug-candidate for the treatment of Alzheimer’s disease.

## Materials and Methods

### Animals

This work was carried out in accordance with the Policies of the French Committee of Ethics. Animal housing facility of the CRCA is fully accredited by the French Direction of Veterinary Services (C 31-555-11, Feb 9, 2011) Animal surgery and experimentation are authorized by the French Direction of Veterinary Services to JML (# 31–122, 2007) and BF (# 31–205, 2011). C57BL/6J 9–12 week old male mice obtained from Charles River were reared in the laboratory breeding facility. They were housed in groups of 3 to 6 per cage and maintained at a constant temperature (21±1°C) with a 12-h light/12-h dark cycle (lights on at 8:00 a.m.). Water and food were available *ad libitum*.

### Surgery and Injections

**Figure 6:**
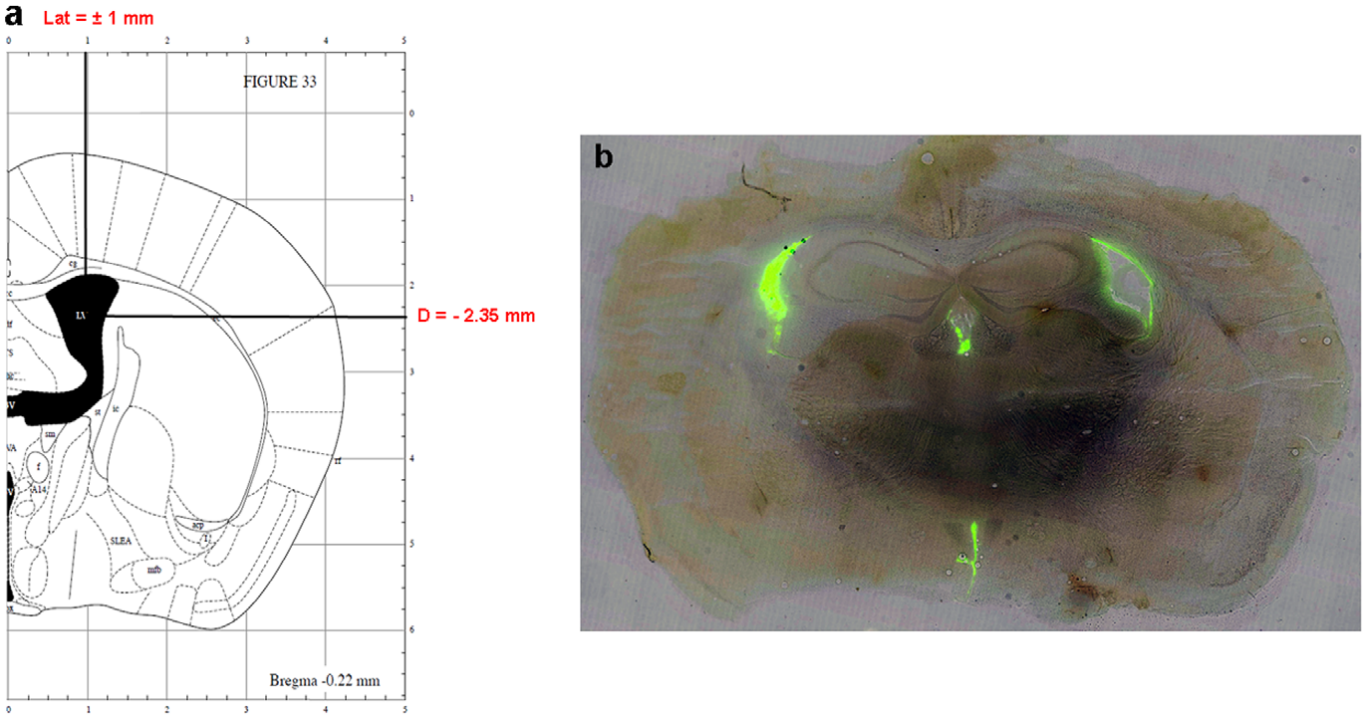
Injection site coordinates (a) and diffusion of fluorescent Aβ_1–42_ (b) in the ventricles (Bregma  =  −1.22 mm).

Mice were anaesthetized with a mixture of chloral hydrate (400 mg/kg, i.p.) (Prolabo) and xylazine (15 mg/kg, i.p.) (Rompun, Bayer Parma) before being placed in a stereotaxic frame (La Précision Cinématographique). Coordinates of the injection site: AP = −0.22 mm from Bregma; L = +/−1 mm from Bregma and D  =  −2.35 mm from skull were chosen according to Franklin and Paxinos [40]. We used fluorescent Aβ to visually check on some mice the presence of Aβ in the lateral ventricles (Figure 6). The skull was drilled and an injector (27 G, 35 mm) was guided successively into the lateral ventricles. After surgery, mice were allowed a 21-day rest.

Aβ_1–42_ amyloid peptide was injected in the lateral ventricles. Intracerebroventricular (icv) injections were made using a Hamilton microsyringe of 5 µL capacity mounted on an automated pump (Razel Scientific Instruments). The injector was connected to the syringe by a flexible polypropylene tube. The entire volume of solution (5 µL) was delivered at the rate of 0.250 µL/min, each ventricle receiving a volume of 2.5 µL. After each injection, injectors were let in place for 10 min in order to avoid flow back of solution. Moreover, a 10 min break was observed after the injection of 1.25 µL. Implantation and injection procedures lasted approximately one hour.

### Amyloid Preparation

**Figure 7:**
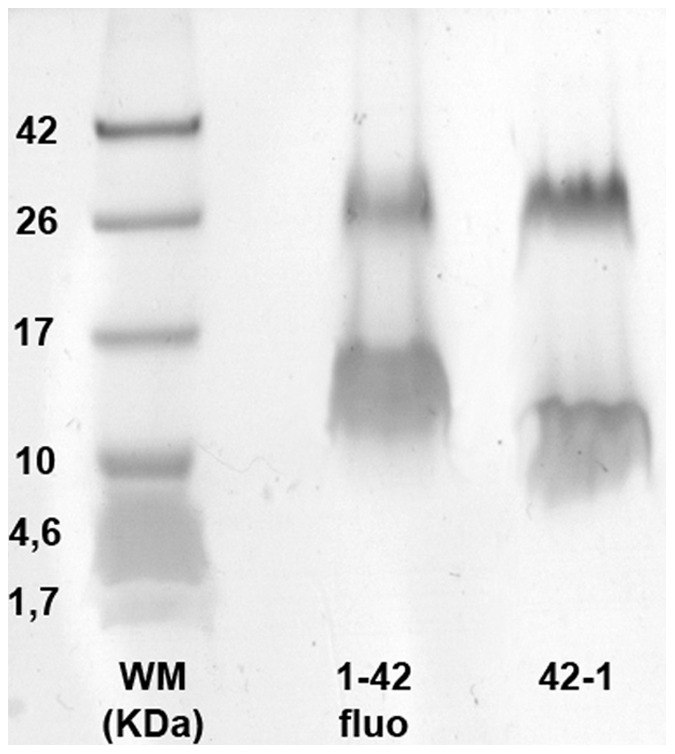
SDS-PAGE electrophoresis of amyloids Aβ_1–42_ (central lane) and Aβ_42−1_ (right lane) without β-mercapto-ethanol. The injected peptidic solutions were identified as trimeric and hexameric oligomer forms.

Aβ_1–42_ fluorescent amyloid peptide was provided as lyophilized white powder by Rpeptide (ref. A-1119-1). The molecular weight for the monomeric form was 4876 Da. This peptide was solubilized in DMSO (Sigma Aldrich, ref. D2438). DMSO was injected directly in the peptide original bottle, using 550 µL of pure DMSO for 0.5 g of peptide. This procedure provided a solution of β-amyloid peptide at a concentration of 2.01×10^−4^ mol/L. Afterwards this preparation was submitted to vortex and sonication to obtain a translucent and homogeneous solution. This solution was divided into aliquots stored up to 3 months away from light at 20°C. Before each experiment, one aliquot was thawed and the aggregation state of the Aβ_1–42_ amyloid solution was checked by gel electrophoresis. This control allowed us to use for all experiments trimeric and hexameric aggregated peptide forms (Figure 7). SDS-PAGE electrophoresis experiments were carried out by using a BioRad system (Mini-PROTEAN Electrophoresis Cell) with 12% acrylamid gel and Tris-Tricine running buffer (Fermentas, ref: B48). Samples were prepared from stock solution and a weight marker from Fermentas (ref: SM1861, range 1.7–40 kDa) was employed to assess weight of different peptide forms. The first tests of gels were conducted with scales up to 250 kDa. Neither oligomers beyond 50 kDa nor aggregated forms were found under these conditions. So the scale was refocused to lower molecular weights to be sure to detect any monomers. Therefore only trimer and hexamer species were found. The Aβ_42−1_ antisense peptide, used in the control experiment, was provided by Bachem (ref. H-3976) and prepared as described above for Aβ_1–42_.

### Drug Treatment

PA1637 was provided by Palumed, clioquinol by Sigma Aldrich (ref. 24880) and memantine was provided by TCI (ref. D3608). Treatment solutions using these three molecules were adjusted to the mean weight of animals.

For oral route administration, PA1637 was dissolved in appropriate volumes of vehicle: methylcellulose (0.6% w/v; Sigma, ref M-0430) and Tween 80 (0.5% v/v; Sigma, ref. P4780) previously heated to 37°C. The stability of PA1637 solutions in that vehicule has been checked over a period of 3 days. No degradation was observed. Before administration to animals, this solution was shaken several times. A 25 mg/kg/day dose was calculated from this solution. 10 µL/g were administered via oral route using a 1 mL syringe and a force-feeding cannula. Mice were treated three times per week for the first two weeks and two times the last week; they received a total of 8 drug doses of 25 mg/kg/dose. In the absence of pharmacokinetics parameters concerning PA1637, this drug treatment regimen was established to obtain a curative effect.

For ip route, clioquinol and PA1637 (provided as powders) were dissolved in appropriate volumes of vehicle: pure polyethylene glycol (PEG400, Sigma Aldrich, ref. P-3265) previously heated to 37°C. Before injection to animals, the solutions were shaken several times. A 25 mg/kg/day dose was calculated from these solutions. Eight injections were made on a 3-weeks period (same schedule as for oral route). For i.p. route, memantine (provided as powder) was dissolved in appropriate volumes of 9% NaCl solution (Sigma, ref. S8776) and polyethylene glycol (PEG400, Sigma-Aldrich, ref. P-3265) (50/50, v/v) previously heated to 37°C. A limpid solution was directly obtained. Before injection to animals, the solutions were shaken several times.

### Binding Constant Determination, Solubility and Stability of PA1637

The binding constant of PA1637 for copper (II) was determined as previously described [15]. PA1637 has a LogD value of 2.5, compared to 3.8 for clioquinol, in octanol/Tris buffer 20 mM with NaCl 150 mM. The water solubility of PA1637 is just below 100 µg/mL. PA1637 is stable for 24 h as powder at 80°C or in different solutions at 37°C (in phosphate buffer at pH 7.4 or in mice blood). The molecule is also stable in HCl 0.1 M for 24 h at room temperature.

### Contextual Fear Conditioning Procedure

Conditioning experiments started 21 days after surgery using the Panlab s.l. Startfear 1.06 system for fear conditioning. A conditioning procedure with a single trial was used in this study. During training, mice stayed in the conditioning chamber for a total of 3 min 30 sec. After a 2 min exploration period, a 85 dB sound (CS) was emitted for 30 sec, and a 0.7 mA foot-shock (US) was superposed to the tone during the last 2 sec. Thirty seconds after the foot-shock, the mouse was gently removed from the chamber and returned to its home cage. Twenty-four hours after the conditioning session, the contextual memory was assessed by introducing the mouse in the conditioning chamber. The behavioral index chosen in this paradigm to quantify the memory of context conditioning is freezing, a species-specific tonic immobility response. Freezing is defined as a tonic immobilization, with total absence of movement except those due to breathing [41]. Freezing scores were assessed during 4 min, no tone or foot-shock being presented to the animal during this test. The occurrence of freezing was scored every 5 sec during conditioning and memory sessions according to the method of instantaneous sampling at fixed time intervals [42]. Data were converted to the percentage of samples scored at freezing during the 4 min context test period.

### Data Analysis and Statistics

Group sizes ranged from 4 to 6 mice. Results in Figures are presented as mean percent times spent freezing. Nevertheless, to satisfy the requirements for the use of ANOVA, mean percent freezing scores (P) were transformed in Q  =  arsin(√P/100). Statistical analyses were performed on the Q variable, using a one-way analysis of variance (ANOVA), or repeated measures ANOVA design for related samples (SYSTAT-11 for Windows). Prior to ANOVA statistics, normality and homoscedasticity were checked for every group. All post hoc comparisons were conducted using the Fisher’s LSD test. α levels were set at P<0.05 for all tests.

### Absence of Confusing Effects of Treatments During Learning Experiments

In the 3 experiments, during the CFC training session the percentage of time spent freezing, summed every 30 sec period remained low (around 30%) and similar among the different groups, then suddenly increased around 70% after the delivery of the electric shock at 2 min 30 sec (PA1637 oral route: F (5,60) = 12.907; p<0.001; clioquinol and PA1637 IP: F (5, 80) = 28.595; p<0.001; memantine IP: F (5, 55) = 58.294; p<0.001) with neither treatment (PA1637 oral route: F(2,12) = 0.611; p = 0.559; clioquinol and PA1637 IP: F(3, 16) = 2.335; p = 0.113; memantine IP: F(2, 11) = 3.450; p = 0.069) nor period x treatment significant effect (PA1637 oral route: F(10,60) = 0.839; p = 0.593; clioquinol and PA1637 IP: F(15, 80) = 0.755; p = 0.722; memantine IP: F(10, 55) = 1.543; p = 0.149). These results revealed that whatever treatment was applied, reactivity and short term memory of mice were not impaired during conditioning since mice showed similar initial levels of freezing and reacted similarly to the unconditional stimulus, displaying a large increase of freezing in response to the electric shock.
